# The association between functional movement and overweight and obesity in British primary school children

**DOI:** 10.1186/2052-1847-5-11

**Published:** 2013-05-15

**Authors:** Michael J Duncan, Michelle Stanley, Sheila Leddington Wright

**Affiliations:** 1 Coventry University, Coventry, UK; 2 Department of Biomolecular and Sports Sciences, Coventry University, Coventry, CV1 5FB, UK

**Keywords:** Functional movement screen, Movement patterns, Mechanics, Weight status

## Abstract

**Background:**

The purpose of this study was to examine the association between functional movement and overweight and obesity in British children.

**Methods:**

Data were obtained from 90, 7–10 year old children (38 boys and 52 girls). Body mass (kg) and height (m) were assessed from which body mass index (BMI) was determined and children were classified as normal weight, overweight or obese according to international cut offs. Functional movement was assessed using the functional movement screen.

**Results:**

Total functional movement score was significantly, negatively correlated with BMI (P = .0001). Functional movement scores were also significantly higher for normal weight children compared to obese children (P = .0001). Normal weight children performed significantly better on all individual tests within the functional movement screen compared to their obese peers (P <0.05) and significantly better than overweight children for the deep squat (P = .0001) and shoulder mobility tests (P = .04). Overweight children scored significantly better than obese in the hurdle step (P = .0001), in line lunge (P = .05), shoulder mobility (P = .04) and active straight leg raise (P = .016).

Functional movement scores were not significantly different between boys and girls (P > .05) when considered as total scores. However, girls performed significantly better than boys on the hurdle step (P = .03) and straight leg raise (P = .004) but poorer than boys on the trunk stability push-up (P = .014).

**Conclusions:**

This study highlights that overweight and obesity are significantly associated with poorer functional movement in children and that girls outperform boys in functional movements.

## Background

Childhood overweight and obesity persists as a major worldwide health issue [1,2]. A number of studies have documented influences such as sedentary behaviour and nutritional habits on weight status but few have examined the structural and functional limitations of excess weight [3,4]. These studies have predominantly been in adult samples and few data have been reported for pediatric populations [3,5]. This is somewhat surprising as children display alterations to their functional movement as a consequence of excessive weight [5] which can impede daily physical activity level and limit functional performance [5,6]. Suboptimal movement patterns resulting from overweight and obesity in childhood may lead to orthopaedic abnormality in later life and inability to complete tasks of daily living. It has therefore been suggested that minimising impaired movement patterns evident in obese and overweight children should be treated at the earliest opportunity [7]. However, few studies appear have examined these associations in children. Studies of gait in pediatric samples [8,9] have identified differences between overweight/obese and normal weight children. These studies documented increased joint moments for overweight children compared to normal weight children [9] and larger joint powers in obese children [8] during walking at 2 different cadences. These studies suggested that the kinematic changes seen in overweight children may have long-term orthopaedic implications [8] and that the lower joint powers seen in obese children contribute to difficulty performing locomotor tasks and potentially decrease motivation to exercise [9]. Moreover, several studies have identified that, although different to functional movement, fundamental movement skills, locomotor (running, hopping, jumping) and object-control (catching, throwing) skills are negatively influenced by overweight [10-12]. It can be argued that good functional movement is needed in order for children to perform these fundamental movement skills [10] and prior studies have consistently reported poorer fundamental movement skills in overweight and obese children [10-12] indicating that childhood obesity might have adverse effects on gross motor development. It is also important to note that the term ‘FMS’ has been used by different authors to represent ‘fundamental movement skills’ e.g. [10] or ‘functional movement skill’ e.g. [13]. The use of this abbreviation can therefore cause confusion, particularly because functional movement skill is different to, but overlaps with ‘fundamental movement skills’. Where fundamental movement skills form the prerequisites for sport competence and other forms of PA [14,15], functional movement skill refers to the underlying movement patterns which underpin performance in all other movements [13]. In the context of the current paper, the term ‘FMS’ will be used in reference to the ‘Functional Movement Screen’.

Other work [3] has more specifically examined the association between Body Mass Index (BMI), physical activity and functional movement by employing the Functional Movement Screen (FMS) in a sample of 58 children. The authors reported that poorer functional movement was associated with higher BMI and lower levels of physical activity. The FMS, employed in the aforementioned study, consists of a series of 7 fundamental movements tests designed to categorise functional movement patterns [13,16,17]. The 7 movement tests use a variety of positions and movements closely related to normal growth and development and it is conceptualised that fundamental movements, such as those tested by the FMS, operate as the basis of more complex movement patterns used in common daily activities [4]. As such the use of the FMS offers a practical way by which to evaluate functional movement patterns. Each of the 7 tests can be scored and presented individually or a total composite score can be generated which provides an indication of functional movement. In the case of the research by Duncan and Stanley [3] only a total FMS score was examined, thus limiting the conclusions that could be made regarding the relationship between obesity and functional movement in children. Recent research by Schneiders et al. [4] presented data from each of the 7 FMS tests on young adults but suggested that future research was needed employing this approach with different populations including children. In particular, no study to date has examined potential differences in functional movement patterns between overweight and obese children (instead preferring to group children as overweight/obese). Moreover, studies presenting data relating to FMS testing in children have used a total FMS measure. As prior studies have identified no difference in total FMS but differences in individual FMS tests [4], it is important to also examine potential differences in scores on each of the FMS tests to make meaningful comparisons between groups and to target interventions more effectively [4] based on a better understanding of how gender and/or weight status might influence the tests within the FMS which will require different contributions of mobility, strength and flexibility.

As data examining functional movement in children and particularly that examining the effect of weight status on functional movement is lacking, the aim of this study was to examine relations between functional movement patterns and weight status in primary school children.

## Method

### Participants

Following institutional ethics approval, ninety children (38 boys and 52 girls, 86% Caucasian, 12% south Asian, 2% Black) from a primary school in Central England volunteered and returned signed parental informed consent forms to participate in the study. Children were aged 7-10 years (mean age ± SD = 9.6 ± 1.4 years). Participants were included if they were ‘apparently healthy’ children aged 7 to 10 years. Exclusion criteria included; the use of a mobility aid or prophylactic device (e.g., knee brace), if they had a musculoskeletal impairment or injury or head injury (<6 weeks) which was likely to affect their motor performance. Children who were diagnosed with any form of developmental disorder likely to influence motor performance were also not included (i.e., developmental coordination disorder, dyspraxia, dyslexia, Asperger’s syndrome and autism).

### Procedures

#### Anthropometry

Body mass (kg) and height (m) were measured to the nearest 0.5 kg and 0.5 cm respectively, using a stadiometer and weighing scales (Seca Instruments, Germany, Ltd). Children were assessed in bare feet and lightly clothed (in their Physical Education kit) and measurements were taken by anthropometrists accredited by the International Association for the Advancement of Kinanthropometry (ISAK) and using the standard ISAK protocol for such measurements. From this, body mass index was determined as kg/m^2^. Children were classified as normal weight (63.7%, n = 58), overweight (15.5%, n = 14) or obese (20.8%, n = 18) according to IOTF criteria [18].

#### Functional movement assessment

The Functional Movement Screen™ (FMS™) is a pre-participation screening tool which evaluates the Fundamental Movement Patterns that underpin performance of all movement [13,17]. The FMS consists of seven tests; Deep squat, in-line lunge, hurdle step, shoulder mobility, stability push-up, rotational stability and active straight leg raise which challenge an individual’s ability to perform basic movement patterns that reflect combinations of muscle strength, flexibility, range of motion, coordination, balance and proprioception [13,17]. The FMS was administered by a trained rater using standardised procedures, instructions and scoring processes [13,17]. Each participant was given 3 trials on each of the seven tests in accordance with recommended guidelines [13,17]. Each trial was scored from 0 to 3 with higher scores reflecting better functional movement. In regard to the criteria for scoring on the FMS, a score of ‘3’ is awarded for perfect execution of the movement, ‘2’ for execution that demonstrates compensation and less than perfect form, ‘1’ where there is inability to complete the movement pattern because of stiffness, loss of balance or another difficulty and a score of ‘0’ is awarded if there is pain when performing the movement. Comprehensive instructions for each movement are also provided elsewhere [13,17]. A pilot study was also conducted with 15 participants to determine test re-test (two-week) agreement between the investigator conducting the FMS rating in the present study. Kappa values revealed excellent test re-test agreement for total composite score and individual FMS tests (Kappa = 0.97 to 1). Although, independent research examining the validity of the individual tests within the FMS is not available in children, studies have evidenced concurrent validity of tests within the FMS with measures of total body performance [19] and have established biomechanical differences in scoring categories for the FMS in the deep squat [20]. Okada et al. [19] recently reported significant relationships between FMS scores on the hurdle step, shoulder mobility, stability push-up and rotary stability tests with backwards overhead medicine ball throw performance. As the backwards overhead medicine ball throw has been established as a test of integrated and dynamic total body movement [21,22], the relationships reported by Okada et al. [19] with FMS tests of mobility, stability and muscular strength evidence logical and concurrent validity of these FMS tests in the context of total body movement. Other research by Butler et al. [20] demonstrated that individuals who score in different categories in the deep squat within the FMS presented different in kinematically determined movement mechanics evidencing validity of this test within the FMS as a measure of movement quality.

For each test, the highest score from the three trials was recorded and used for analysis. These scores were also summed to generate an overall composite FMS score with a maximum value of 21 and in accordance with recommended protocols [4,13].

In the present study both the composite FMS score and individual scores for each component of the FMS were used. This is consistent with previous research with young adults [4] and children [23]. The composite FMS score provides a holistic evaluation of an individual’s functional movement [13,17]. However, individual scores for each of the FMS tests are needed to determine an individual’s consistency of movement, to determine whether there is a specific movement dysfunction, to pinpoint future intervention [13,17] and to evaluate movement patterns between different groups (e.g., boys vs. girls) [23].

#### Data analysis

As data did not meet the requirements for parametric statistical analysis, non-parametric tests were used for analysis. Relationships between total FMS score and BMI were analysed using Spearman’s rank order correlation. Differences in scores for each test and the total composite score between gender groups were assessed using the Mann–Whitney U test and any differences in scores for each test and the total composite score between normal weight, overweight and obese children were examined using the Kruskal-Wallis test. Where any significant differences were found post-hoc analysis employing the Mann–Whitney U test with Bonferroni correction was used to determine where these differences lay [24]. The statistical package for social sciences (SPSS, Version 18) was used for all analysis and statistical significance was set a priori as P = .05.

## Results

Total FMS score was significantly, negatively correlated with BMI (rho = −.572, P = .0001). Mean ± S.D. of total FMS scores when split by gender and weight status groups is presented in Table 1. Results indicated no significant difference in total FMS score between boys and girls (U = 884.0, P > .05). However, total FMS score was significantly different between normal weight, overweight and obese children (H = 47.8, P = .0001). Post hoc testing identified that total FMS scores were significantly higher in normal weight compared to overweight children and obese children and was also significantly higher in overweight compared to obese children (all P = .01 or better).

**Table 1:** Mean ± (S.D.) of total FMS scores for gender and weight status groups

	**Normal weight (n = 58)**	**Overweight (n = 14)**	**Obese (n = 18)**	**Total (according to gender)**
Males (n = 38)	14.6 (2.4)	12.6 (0.9)	8.2 (1.3)	12.7 (3.4)
Females (n = 52)	14.7 (2.4)	12 (0.5)	10.4 (1.4)	13.6 (2.7)
Total (according to weight status)	14.7 (2.4)	12.2 (.07)	9 (1.7)	13.2 [3]

**Figure 1:**
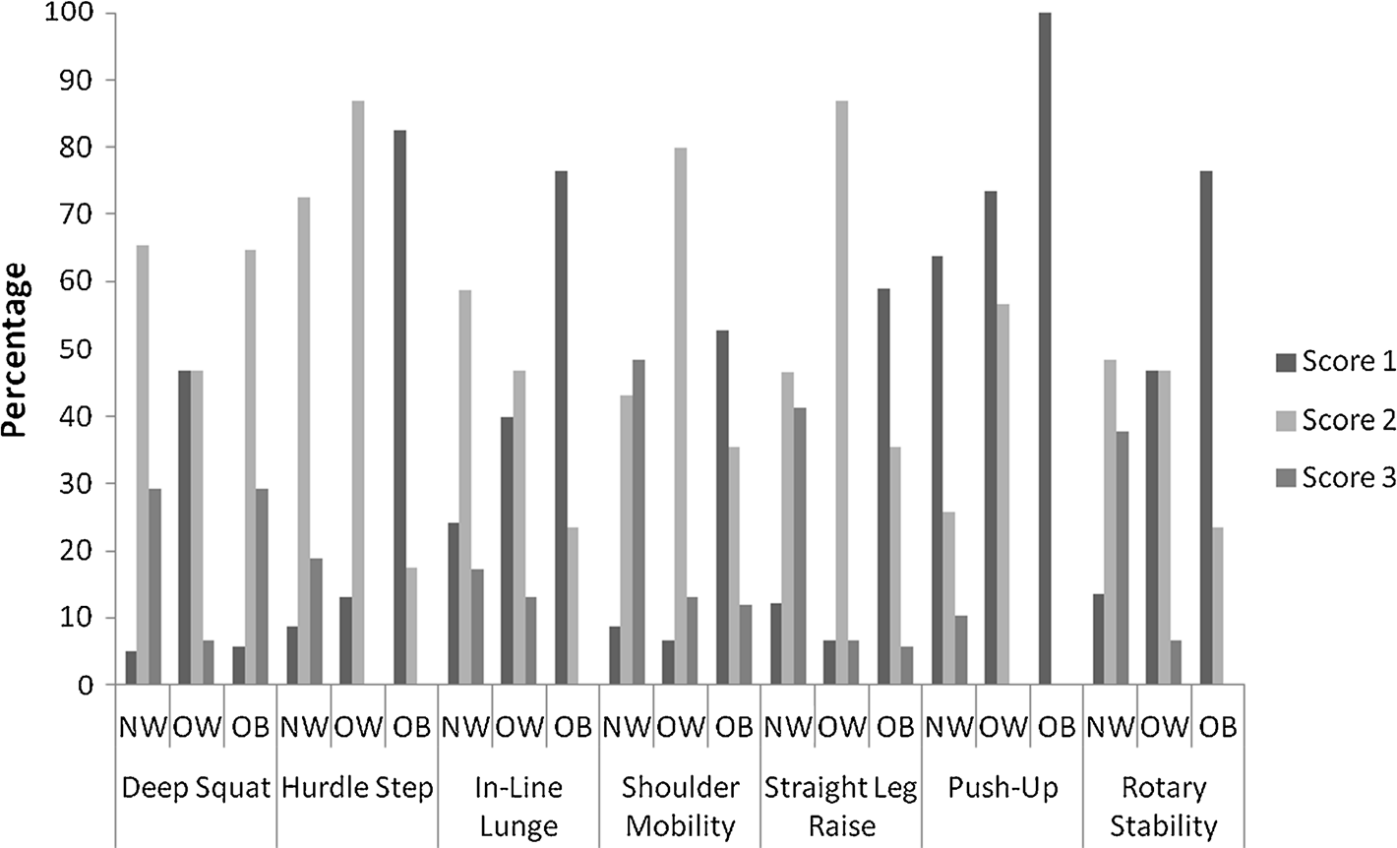
Score distributions for each individual FMS test between normal weight (NW), overweight (OW) and obese (OB) children (a score of 0 was not recorded for any participants).

**Figure 2:**
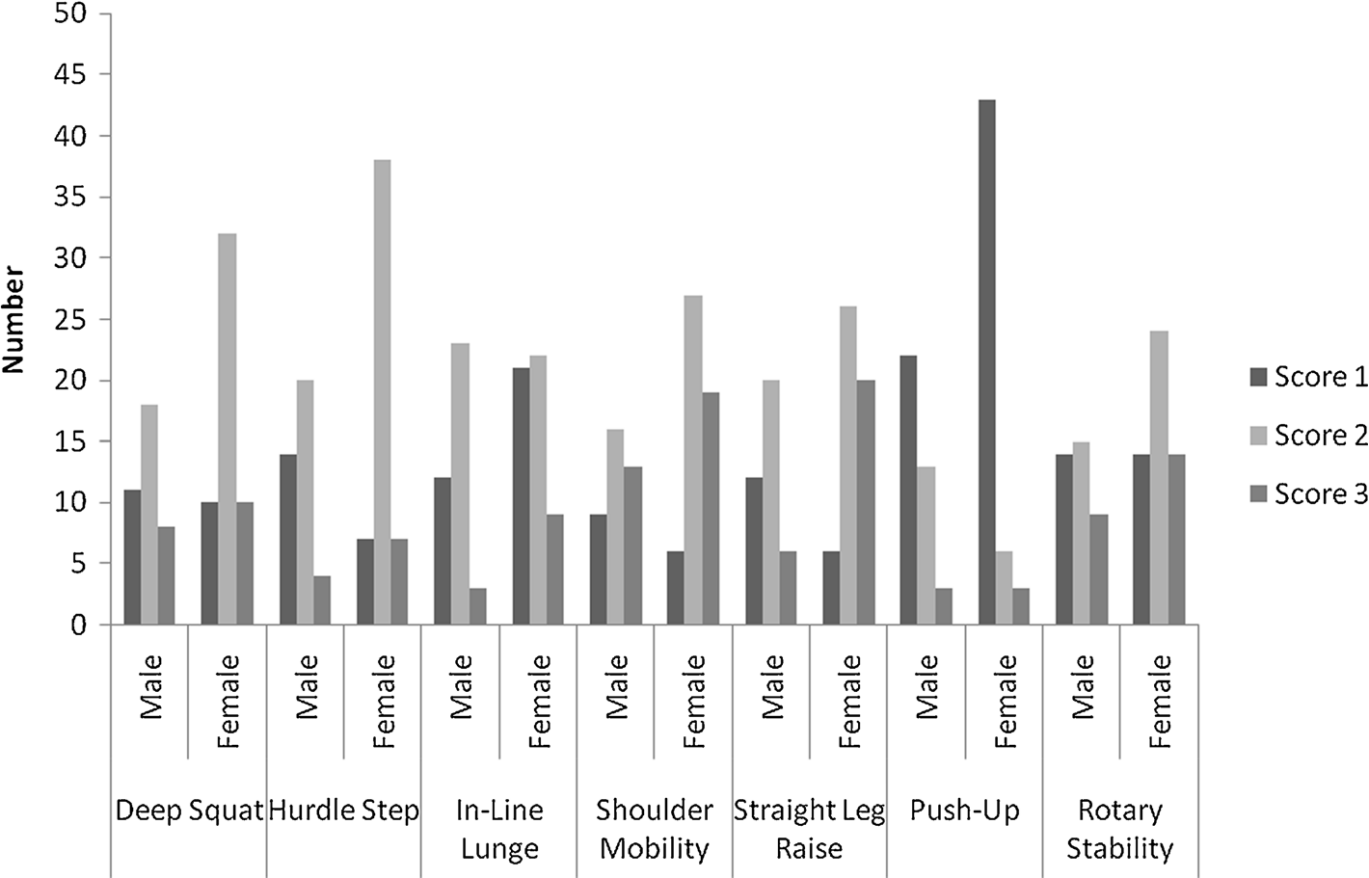
Score distributions for each individual FMS test between boys and girls (a score of 0 was not recorded for any participants).

The distribution of scores for the different FMS tests are presented in Figure 1 for weight status groups and Figure 2 for gender groups. In regard to differences in FMS test scores between normal weight, overweight and obese children, results from statistical analysis indicated significant differences between normal weight, overweight and obese children in all the tests of the FMS (P = .001). However, the pattern of scoring differed depending on the particular test within the FMS between weight status groups. In all cases normal weight children were more likely to score a ‘2’ or a ‘3’ than their obese peers (all P = .005 or better). However, normal weight children only scored significantly better than overweight children for the deep squat (P = .0001) and shoulder mobility tests (P = .04). In regard to comparisons between overweight and obese children, post hoc analysis indicated that overweight children scored significantly better in the hurdle step (P = .0001), in line lunge (P = .05), shoulder mobility (P = .04) and active straight leg raise (P = .016). Of particular note, no obese children obtained a score of ‘3’ in the hurdle step, in line lunge, rotary stability or trunk stability push up.

In regard to gender (See Figure 2) there were significant differences for the hurdle step (*U* = 763, P = .03), straight leg raise (U = 668, P = .004) and trunk stability push-up (*U* = 753.5, P = .014).

## Discussion

This study adds to the literature in the area by providing data examining the association between weight status and functional movement screen scores in children. Results of this study suggest that functional movement is related to weight status with functional movement being poorer in children classified as obese across all 7 tests in the functional movement screen, compared to their normal weight peers. In this respect the results of the present study are confirmatory of prior work [3] that reported total FMS score to be significantly and inversely related to BMI in a group of 10–11 year old children. However, in contrast, the present study clearly builds on the work presented by Duncan and Stanley [3] by presenting data across the individual tests within the Functional Movement Screen. This is important as individual scores on the 7 tests within the FMS as these are purported to relate to the range of activities involved in human tasks of daily living [4] and represents how the present study adds to the area. While the use of a composite score of functional movement, such as that by prior research [3] provides a holistic indication of the total quality of an individual’s or group’s functional movement. The use of a composite score solely, as is the case in the prior work by Duncan and Stanley [3], limits the diagnostic value of the Functional Movement Screen because it does not enable researchers or practitioners to understand whether overweight status had a specific impact on all functional movement skill. Nor does it highlight or whether there was patterning to the effect that overweight and obesity might have on specific functional movements (e.g., those requiring strength over balance, upper body over lower body functional movements). As such, prior work presenting the association between functional movement and weight status in children [3] might be considered limited in comparison to the data presented int he present study. Similar data have also been reported for a sample of young adults by Schneiders et al. [4] but without examining the effect of weight status. The data presented in this manuscript might therefore be considered as also extending current knowledge on expected ranges of functional movement performance in children.

This study also supports prior research in adults which has suggested that excess weight results in functional limitations [5,25] and assertions that children display altered functional movement as a consequence of excess weight [5,6]. However, in the context of the present study and population examined, it is possible that functional limitation may have existed prior to overweight/obesity. Therefore, it may be that functional limitation may lead to children being less physically active, gaining fat mass and will not develop the Fundamental Movement Patterns that underpin performance to the same level of mastery as children who do not have a functional limitation [26]. In the same way, children who are not functionally limited may more engage in more daily physical tasks and thus, engage in more regular practise of the Fundamental Movement Patterns that underpin performance. These children will consequently become more proficient in performance of Fundamental Movement Patterns resulting in greater physical self efficacy, increased likelihood of participation in physical activity and lower likelihood of unhealthy weight. This suggestion is conjecture although prior studies have identified that greater self-efficacy is associated with better functional performance and less functional limitation in older adults [27] and recent research has highlighted that movement training and physical activity programmes are effective in enhancing fundamental movement skills in children [28]. Although fundamental movement skills are discrete from functional movement, functional movement skills are the prerequisites for the performance of fundamental movement skills.

To the authors’ knowledge this is the first study to examine associations between weight status and the individual tests within the FMS in a pediatric population. The data presented here are however important as they highlight those children who are overweight and obese present maladaptive movement patterns needed to accomplish tasks of daily life. Moreover, in the context of the current study the condition of overweight versus obese cannot simply be equated in terms of FMS outcomes. The results of this study suggest a clear differentiation in the pattern of scoring for functional movement between normal weight, overweight and obese children. Obese children evidenced significantly poorer functional movement scores on all the tests within the FMS in comparison to normal weight children. However, overweight children only performed more poorly than their normal weight peers in the deep squat and shoulder mobility tests. The subsequent poorer performance by obese children compared to their overweight peers in the hurdle step, in line lunge, shoulder mobility and active straight leg raise tests might also suggest a graded deterioration in functional movement in normal weight compared to overweight and overweight compared to obese children. As this is the first study to compare individual test scores in children across normal weight, overweight and obese children (as opposed to combining overweight and obesity in one category) further research is needed to determine if this represents a dose–response in relation to the effect of excess body mass on functional movement.

Over time, these movement patterns coupled with the effect of excess weight on joint loading are likely to lead to orthopaedic abnormality in later life [6]. Moreover, such suboptimal movement patterns can prevent individuals from undertaking health enhancing physical activity [6].

Mechanisms for the impact of overweight and obesity on functional movement. Have been proposed by prior authors [6] but these are based on adult studies due to the dearth of data in children. Overweight and obesity has been suggested to lead to alterations in the musculoskeletal system that place overweight individuals at higher risk of musculoskeletal pain [6]. This increased musculoskeletal pain then results in restricted range of movement. Some studies in children and adolescents [28,29] have evidenced changes in foot structure and plantar pressure distribution, as a result of structural adaptation to excess weight, which was suggested to lead to functional movement complications. Research with older adults [30] has also highlighted that a BMI of 30 kg/m^2^ or greater is significantly associated with functional limitation. The data presented in the current study would, at least in part, support this assertion in children. The suggestion that there is a reciprocal relationship between functional movement/motor competence, physical activity and weight status is not new and the results presented here align with the conceptual model previously presented by Stodden et al. [31]. However, other research with older adults has additionally suggested that the ratio of fat free mass (FFM) to body mass is important as individuals with higher FFM are more likely to be functionally proficient than those with lower FFM [32]. Future research would therefore be welcomed in this area but using pediatric samples.

Despite this, the data presented here support the need for interventions to improve functional movement in British children generally but those who are overweight and obese specifically. It is also important to note that although the children in the present study had been familiarised with the movements involved in the FMS, it may be that there are practice effects in the FMS. Studies have yet to ascertain whether this is the case.

When comparing these study results in relation to gender, one other study to date [23] has reported specifically on the use of the tests within the Functional Movement Screen in a pediatric population. In a relatively small sample of 39 middle school children, Burton et al. [23] reported that higher total FMS scores in girls compared to boys were as a result of better performance in the deep squat, in line lunge, straight leg raise and shoulder rotation. The range of total FMS scores reported in their study [23] are similar to those documented in this study. However, no overall gender difference was reported here, contrasting the total FMS results reported by Burton et al. [23]. Despite this, the results of the present study would agree with assertions made by Burton et al. [23] that girls outperform boys in functional movements requiring hamstring flexibility and balance whilst boys outperform girls in tests where muscular strength is required. However, these results also agree with data presented by Duncan and Stanley [3] which reported no gender differences in total FMS scores of 10–11 year old children. One potential explanation for the gender differences in particular functional movements reported in the present study has been children’s physical activity socialization [15]. Opportunities to practice functional movements related to strength performance tend to be higher in boys, since significant others (parents, peers) may often prioritise participation in these activities for boys whereas girls tend to more regularly participate in sports such as dance or gymnastics [33,34] which refine movement and postural control skills such as balancing [35-37]. In such a case the movements required to perform such activities would best relate to the hurdle step and straight leg raise tests within the functional movement screen as used in the present study and may, at least in part, explain the gender differences reported in this study. Similar to the present study, Schneiders et al. [4] reported gender differences of the same magnitude in the straight leg raise and trunk stability push-up in a population of young adults.

Although there are various measures that have been used in studies to quantify fundamental movement skills [33,34,36,37], the functional movement screen employed in this study provides a way to assess the quality of specific movement patterns that relate to movement rather than the ability to perform a function (e.g., to throw a ball at a target as an evaluation throwing skill). Moreover, although the FMS as used in the present study has most readily been used with athletic groups [13,17], the FMS was conceptualised to provide a practical screen to identify biomechanical deficits in fundamental movement that may limit human performance [13,17]. Research has also examined functional movement using the FMS in young adults [4], firefighters [38], following worksite yoga intervention [39] and in children [23]. Thus, the use of the FMS appears to a practical and participant friendly way by which to evaluate movement in children. However, there may be remit for future research to specifically validate the FMS with performance of everyday life activities in a pediatric population. Furthermore, we also acknowledge that the FMS as used in the current study may not be the best option to evaluate movement quality in children in a field setting.

It is also important to note that the results presented here do not necessarily suggest a clinical need for those children exhibiting poor functional movement at their present age. However if those children who exhibited suboptimal movement patterns persist into adolescence and adulthood and are coupled with overweight/obesity this may lead to further musculoskeletal problems of clinical significance including chronic pain, knee osteoarthritis and early hip replacement (See [5] for a review) at a later stage.

There are also potential confounding variables that may have influenced the results presented in the present study. Habitual physical activity may represent one such confounding variable and the lack of assessment of this variable should be considered as a limitation in the current study. Other individual variables such as socio-economic status and ethnic group may also potential influence functional movement and were also not accounted for in the current study. The present sample did not have a sufficient balance of ethnic groups nor was it drawn across multiple socio-economic status groups for these confounding variables to be accounted for. It may also be important for future studies to assess or control for such confounders when considering the impact of overweight/obesity on functional movement in children. Furthermore, longitudinal designs would be welcome to understand whether overweight/obesity leads to a lack of physical activity and subsequent poorer functional movement or whether suboptimal functional performance actually restricts ability to engage in health enhancing physical activity and leads to subsequent unhealthy weight gain. This exploratory study is also limited by a small sample size and larger scale studies would be welcome to verify the claims made here. In addition, cause and effect in relation to weight status and functional movement could not be determined in the present sample.

## Conclusions

The results of this study build on prior studies on this topic by highlighting that overweight and obesity are significantly associated with poorer functional movement in British children compared to normal weight children. Presenting individual FMS scores alongside the total composite score also identified that normal weight children outperformed their overweight peers on the deep squat and tests of shoulder mobility and overweight children outperformed obese children in functional movements requiring hamstring flexibility, shoulder mobility and balance.

These results also highlight that girls outperform boys in functional movements requiring hamstring flexibility and balance whilst boys outperform girls in tests where muscular strength is required. Further research is needed which evaluates interventions to reduce overweight and obesity and/or improve functional movement in children.

## Competing interests

The authors declare no competing interests.

## Authors’ contributions

MD designed the study, Assisted in data collection, performed statistical analysis and wrote the manuscript. MS Assisted in data collection and writing the manuscript. SLW Assisted in data collection and writing the manuscript. All authors read and approved the final manuscript.

## Pre-publication history

The pre-publication history for this paper can be accessed here:

http://www.biomedcentral.com/2052-1847/5/11/prepub
